# Supplementary material for: Effects of environmental awareness training and environmental commitment on firm’s green innovation performance: Empirical insights from medical equipment suppliers
Source: PLoS One. 2024 Mar 5;19(3):e0297960. doi: 10.1371/journal.pone.0297960 (PMC10914289; doi:10.1371/journal.pone.0297960)
Supplement: Attachment — Submitted filename: Dr Jubril PONE Review1.docx [file pone.0297960.s001.docx]

**Full Title:**

Effects of environmental awareness training and environmental commitment on firm’s green innovation performance: Empirical insights from medical device suppliers

**Short Title:**

Environmental awareness training and environmental commitment on firm’s green innovation performance

Reviewer(s)' Comments to Author:

Recommendation: Major Revision

Comments:
The basic idea of the paper is probably nice, The aim of this paper explores the cause and effect relationship of environmental awareness training (EAT), environmental knowledge exchange activities (EKEA), and environmental commitment (EC) on green innovation performance (GIP).Research investigating the effectiveness of environmental education programs and initiatives aimed at promoting the exchange of environmental knowledge. To bridge the research gap, this study utilized the survey data from Chinese medical device manufacturers to verify the research assumptions. Focus group discussions with five medical equipment suppliers were undertaken, and survey data from 285 medical equipment manufacturing companies in China analyzed with a confirmatory factor analysis using AMOS software techniques. Lastly, paper utilized SPSS and Process software to examine the correlation between variables and verify the proposed research hypothesis.

The paper shows excellence in novelty and originality, and the findings have important implications for environmental awareness training that plays a crucial role in promoting green innovation performance,and are clearly presented with assurance of correctness. Also, the study has the potential to influence further research in the field of environmental awareness training and innovation performance. However, there are still some shortcomings. Specific comments are listed below.

**1. Introduction**
The introduction has some strengths, such as providing a clear overview of the topic and highlighting the significance of green innovation performance (GIP).The study is unable to come up with a specific problem from the introduction part. However,there are some weaknesses in the structure and writing of the introduction.

- Firstly, the introduction could be more concise and focused,it could be trimmed down to make the introduction more efficient. Secondly, the introduction could benefit from clearer transitions between ideas. Some of the paragraphs feel disconnected from each other, which makes it harder for the reader to follow the argument.

- Finally,there could be more attention to the flow of the introduction. It might be more effective to start by introducing the problem of GIP firm adoption and the resistance it faces before going into the background information. This would make the introduction more engaging and help to create a sense of urgency around the research question, the introduction could be more effectively set up the rest of the paper.

2. **Literature Review and Hypotheses Development**
   - The title of the second part should be "Literature Review" instead of "Theory and hypotheses development".

   - The paper proposes using the Knowledge Based View (KBV) as an underpinning theory for the study. While KBV is a comprehensive framework, the introduction does not specify the limitations of the model, and whether it has been sufficiently validated in the context of GIP firms.

   - The paper proposes investigating environmental awareness training, environmental knowledge exchange activities, and environmental commitment along with the functional elements of KBV.While these are important aspects to consider, the introduction does not provide a clear rationale for why these dimensions are critical, and how they integrate with KBV to create a more robust model for this study.However, the introduction does not explain the rationale for why this integration is necessary or how it contributes to the research objectives.

- Does the paper demonstrate an adequate understanding of the relevant literature in the field and cite an appropriate range of literature sources?Is any significant work ignored? The authors does not provide an overview of the existing literature, which could be more systematic. Even thou am somehow a supporter of tabular literature reviews that illustrate the respective research focuses. This could also be done here if authors feel like. All in all, there has been much more research on GIP than what is presented here. Authors might think about integrating more research perspectives:It appears suitable overall,but the literature is then very focused on it.

3. **Methodology**

-Is the paper's argument built on an appropriate base of theory, concepts, or other ideas?  Has the research or equivalent intellectual work on which the paper is based been well designed?  Are the methods employed appropriate?: The hypothesis development is rather limited.

- There is a lack of a truly informed reflection on the theories. All in all, there is no reference to young consumers in the entire theoretical foundation. The fact that the empirical study itself was conducted with "young tourists" is not sufficient.

- What sample techniques does the authors use for this study, the entire sampling process, as well as the data cleaning process, are somewhat opaque.

4. **Results**

- Large sample size: This paper collected data from only 285 medical equipment manufacturing companies in China. Are results presented clearly and analyzed appropriately?  Do the conclusions adequately tie together the other elements of the paper?

- Are results presented clearly and analyzed appropriately?  Do the conclusions adequately tie together the other elements of the paper?: The results are presented clearly and analyzed appropriately. The conclusions adequately tie together the other elements of the paper and are supported by the results and analysis presented. The authors have effectively interpreted the results just need to provided a clear and concise summary of the findings. Overall, the results and conclusions are well-supported and contribute to the overall strength of the paper.

**5. Implications for research**

- Does the paper identify clearly any implications for research? Does the paper bridge the gap between theory and practice? How can the research be used in practice (economic and commercial impact), in teaching, to influence public policy, in research (contributing to the body of knowledge)?

- Are these implications consistent with the findings and conclusions of the paper?: The paper clearly identifies implications for research. The authors was unable to effectively bridged the gap between theory and practice by providing practical applications for the research findings. Moreover, the discussion could benefit from a more in-depth reflection on the theoretical implications of the study. For instance, how do the findings contribute to the existing literature on the use of environmental awareness training and environmental commitment on firm’s green innovation performance in the context of medical devices suppliers? What are the theoretical or practical implications of the newly established relationships in the KBV model?

**6. Discussion of Findings**
- Moreover, the discussion could benefit from a more in-depth reflection on the theoretical implications of the study. For instance, how do the findings contribute to the existing literature on the use of the use of environmental awareness training and environmental commitment on firm’s green innovation performance in the context of medical devices suppliers? What are the theoretical or practical implications of the newly established relationships in the KBV model?
- The study in this paper is limited to medical equipment manufacturing companies in China, so the findings may not be applicable to other user groups.

In summary, although this paper provides valuable insights in examining the effect of environmental awareness training and environmental commitment on firm’s green innovation performance,more work is needed to expand on the literature review, improve the paper structure and writing, and deepen the theoretical contributions.
